# Stability-Focused Nanoparticle Development: Applying Particle Size Stability as a Response Factor in the Design of Experiments

**DOI:** 10.1021/acsomega.5c00613

**Published:** 2025-05-07

**Authors:** Vanessa V. B. Muccelin, Otavio C. Vellozo, Thais L. Valente, Carolina G. Pupe, Edison L. S. Carvalho, Cassia B. Detoni

**Affiliations:** Formulation Technology Research Group, Institute of Pharmaceutical Science, Campus Macaé − Federal University of Rio de Janeiro, Av. Aluizio da Silva Gomes, 50 - Granja dos Cavaleiros, Macaé, Rio de Janeiro 27930-560, Brazil

## Abstract

Particle size stability is an important quality parameter of nanoparticle delivery systems and is usually investigated at the end of formulation development. It has not been used as a response factor in the design of experiments (DoE). This study aimed to evaluate the use of particle size stability as a response factor in a DoE, allowing researchers to obtain knowledge on formulation stability early on during formulation development. Nanostructured lipid carriers loaded with copaiba oil were used as the test formulation. The experimental design consisted of 14 runs, including one central point in sextuplicate, and the data were analyzed using multiple linear regression analysis. By using predetermined parameters of size variation, PdI, and analysis quality, a model that measures stability in days offered statistically significant predictors and enabled formulation optimization. The results demonstrate the applicability of DoE for optimizing nanoparticle formulations based on particle size stability as a response factor.

## Graphical abstract

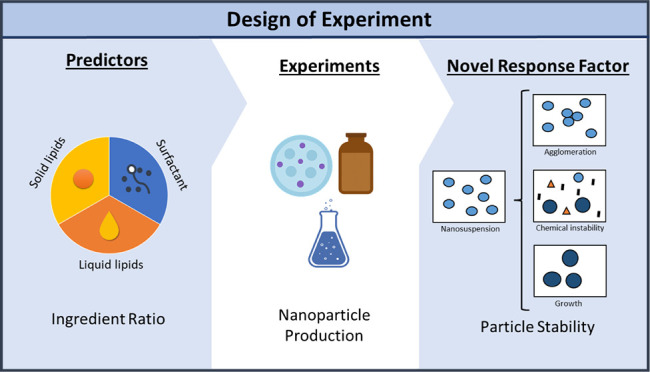

## Introduction

Design of experiments (DoE) is a powerful tool that allows the understanding of multidimensional interaction effects of predictors on response factors in nanocarrier development and optimization. The response factors used in these types of studies usually include entrapment efficiency, drug load, particle size, zeta potential, polydispersity index (PdI), drug release, absorbance/transmittance/turbidimetry, and permeation.^1^

The development of nanomedicines is in its “tuning the engine” phase, concerning product production and clinical translation,^2,3^ and researchers are narrowing down the issues concerning this technology. Stability is among these issues,^3^ −^4,5^ as seen for lipid nanoparticles (LNPs), which are currently in the spotlight as delivery systems for mRNA.^6^ mRNA-LNP formulations, for example, require strict control, including maintenance at relatively low temperatures during transport and storage. Particle size stability is used as a study parameter for these formulations^6^ and can be defined as the preservation of size distribution during storage and/or experiments.^7^ Therefore, an important aspect of development is the selection of proper stabilizers and their proportions^8^ and a potential response factor for DoE using these variables is particle size stability.

Nanoparticle drug delivery system challenges regarding stability include creaming, agglomeration, growth, changes in the crystalline state, and chemical instability.^3,4,8^ Particle size changes as a result of one or more of these phenomena and, therefore, is a quick-to-check physical stability parameter.^8,9^ This parameter is commonly evaluated by dynamic light scattering (DLS),^8,10^ considering changes in Z-average and an increase in the polydispersity index (PdI) above 0.2 or 0.3.^9^ DLS has many advantages, such as practicality, speed, and cost, and it is accepted worldwide by regulatory agencies.

On the other hand, particle size stability measured by DLS does not have a standard quantification method or numerical unit. Many researchers use particle size variation to evaluate nanocarrier stability, but they do not objectively define the determinants of a stable or unstable formulation. Typically, a graph showing low deviation or no statistical difference in size is used when more than one batch has been prepared.^11–13^ In standard formulation development studies, particularly when DoE is employed, batch triplicates are not required and can be time-consuming. Consequently, stability is primarily assessed in the optimized formulation rather than being used as a criterion for its optimization.^13,14^

Documents from the FDA’s “Guidance for Industry” ^15,16^ emphasize the importance of verifying changes to nanomaterial size and size distribution, but do not specify criteria for what constitutes an acceptable variation of size or size distribution.^9^ Therefore, the purpose of this study was to test the applicability of a particle size stability parameter as a response factor in a DoE applied to nanocarrier optimization.

Nanostructured lipid carriers (NLCs) are a type of solid lipid nanoparticle that contain a mixture of solid and liquid lipids in their matrix and surfactants at the water–lipid interface.^17–19^ These particles have been commercially available for over a decade, mainly in oral supplements and topical delivery systems.^17^ Laboratory production of these particles by solvent injection is simple and highly efficient, without the need for sophisticated equipment.^20,21^ Considering these characteristics, we chose NLCs as a model nanocarrier system. To accentuate instability and test the proposed mathematical model, the particles were prepared with a natural seed butter (tucuman butter), which is prone to oxidation,^22^ and a resin oil (copaiba oil) with volatile components.^23^

Central composite design (CCD), a surface response methodology, was used in this study. This design is used to understand the interactions between the selected factors, analyze the effects of factors on responses, and optimize formulations with a minimal number of experiments.^24,25^ The aim of the study was to use particle size stability as a response factor in a DoE, as it has never been used before.

## Results and Discussion

Two possible methods were used to determine particle size stability. In Method 1, the stability of NLC was measured using a size unit (nanometers), considering particle size (*Z*-average) variation after 28 days (Δ*Z* average). In Method 2, the stability of NLC was measured using a time unit (days), in which case stability was defined as the number of days the formulation remained stable. To determine if a formulation was stable at a certain time, Method 2 considered three parameters: *Z*-average variation under 8%, PdI under 0.2, and the sample mean particle size not deviating by more than 5.0% within three readings. After preparation, the particle size of each formulation was measured every week for 28 days. When a formulation was identified as unstable, its stability in days was recorded as the number of days of the prior measurement.

The liquid-to-solid lipid ratio (Liq:So) and the proportion of total lipids to surfactants (TL:Sur) were used as predictors (Table 1). The response factor, particle size stability, was described by either Method 1 or Method 2. Minitab 17 generated 14 experiments, in which reproducibility was tested through a center-point sextuplicate, and the data were analyzed using multiple linear regression analysis.

**Table 1:** Central Composite Design of Surface Response Experiment with Response Values for 14 Experiments

	Predictors		Response fator	
No	Liq:So^a^	TL:Sur^b^	Method 1	Method 2
			Δ*Z*-ave (nm)^c^	Stability (days)^d^
1	2.000	1.000	–12.6	28
2	0.189	1.750	–1.8	21
3	1.250	2.810	1941.3	21
4	0.500	1.000	2.6	21
5	1.250	1.750	–14.1	7
6	1.250	1.750	–2.6	7
7	1.250	1.750	–44.2	14
8	1.250	1.750	–20.9	7
9	1.250	0.690	–53.6	21
10	0.500	2.500	1.0	28
11	1.250	1.750	–86.1	14
12	1.250	1.750	–52.1	14
13	2.000	2.500	–6.0	28
14	2.311	1.750	–58.5	21

a Liq:So is liquid to solid lipid ratio.

b TL:Sur is proportion between total lipids and total surfactants.

c Δ*Z*-ave is z-average variation after 28 days (Method 1).

d Stability in day is the number of days the formulation remained stable (Method 2).

NLCs were prepared using an adaptation of the solvent injection technique (Supporting Information). All formulations were nanometric, with a PdI < 0.2 upon preparation, and are shown as the mean and standard deviation of three readings (Figure 1).

**Figure 1:**
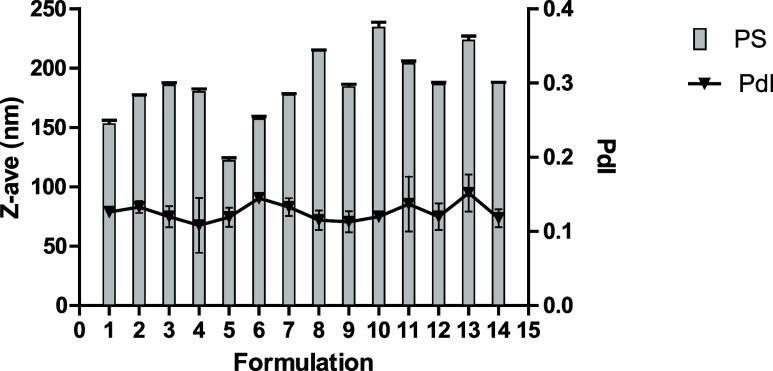
*Z*-average (PS: particle size) and polydispersity index (PdI) of the 14 experiments from the central composite design surface response experiment on the day of preparation, represented by mean and standard deviation as the error bar.

Mean particle size deviation within three readings is used to indicate measurement quality. In the first measurement, all formulations presented a *Z*-average deviation below 5.0% within three readings.

Both methods presented the best fit, with a higher regression coefficient, for the quadratic model. Even so, Method 1 did not show a significant fit (*p* > 0.05) (Table 2). Size instability might have different root causes in each of the 14 formulations, such as chemical instability, Ostwald ripening, or aggregation. Furthermore, a major limitation of DLS is its low resolution in polydisperse systems,^26^ a common characteristic when particles undergo instability phenomena. These aspects explain why Z-average variation (Method 1) is not suitable as a response factor representing particle size stability in a mathematical model.

**Table 2:** Statistical Parameters for Linear and Quadratic Models Used to Analyze Response Factors in Methods 1 and 2

	Response factor	
Parameter	Method 1	Method 2
	Δ*Z*-ave (nm)^a^	Stability (days)^b^
*R*^2^ linear model^c^	27.72%	3.24%
*R*^2^ quadratic model^c^	60.61%	81.37%
Adjusted *R* ^2^ quadratic model^c^	35.99%	69.72%
*p-*value quadratic model^d^	0.088	0.001
*F*-value quadratic model	3.34	16.77
SD quadratic model^e^	421.42	4.19

a Δ*Z*-ave is z-average variation after 28 days (Method 1).

b Stability in days is the number of days the formulation remained stable (Method 2).

c Regression coefficient (*R* ^2^) indicates the model’s goodness of fit, where values closer to 1 represent better predictive accuracy.

d *p*-value (*p* < 0.05) suggests statistical significance, with lower values indicating a significant deviation from random variation.

e Standard deviation (SD) reflects the dispersion of the model’s predictions, with lower values indicating better precision. Model selection was based on the best fit for each response factor.

For Method 2, the *F*-value (16.77) and *R* ^2^ (81.37%) indicate that the quadratic model accurately describes the stability of the NLC. Therefore, the response factor found to be significant (*p* < 0.05) was particle size stability measured in days. The model quality is consistent with CCD models established for lipid-based nanocarriers in previous studies.^24,25^ The results showed that both predictors had a significant effect on particle size stability (*p* = 0.003) (Supporting Information 1 and Table S5).

The contour graph (Figure 2) presents three zones of predicted particle size stability. In zone 1, using higher amounts of solid lipids stabilizes formulations with a lower surfactant proportion. A higher liquid lipid content results in stability with either a high TL:Sur ratio (predominance of lipids) or a low TL:Sur ratio (predominance of surfactants), but not intermediate proportions, as seen in zones 2 and 3, respectively.

**Figure 2:**
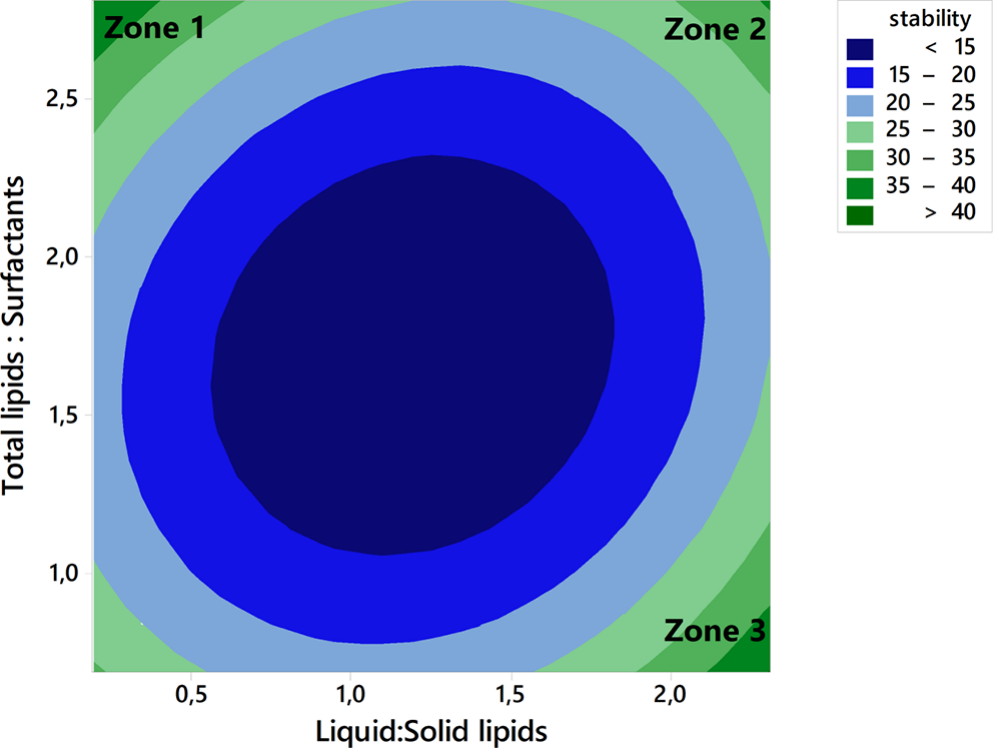
Contour graph of polynomial quadratic model using method 2, in which the response factor is particle size stability measured in days. {Dark green areas indicate high particle size stability and are identified as zones 1, 2, and 3}.

Zone 1 indicates that by increasing the solid lipid content, NLC formulations become more stable. This can be explained by the fact that the solid lipid might function as a pharmaceutical thickener for the dispersed phase. The higher viscosity of the internal phase enhances stability, preventing coalescence in dispersed systems, as happens in conventional oil-in-water (o/w) biphasic systems.^27^ Souto and Muller (2005) also described that the liquid lipid content in the internal phase of NLCs might cause aggregation of the particles.^28^

Higher quantities of lipids in relation to surfactants resulted in higher particle size stability, as observed in zone 2. For this experiment, the surfactant quantity was fixed at 0.08 g mL^–1^ and the ratio at 1:1. This pre-established condition is the main reason for the observed results. At this concentration of surfactants, low quantities of lipids lead to an excess of surfactants. The excess surfactant results in lower stability. A similar observation was made by Pezeshki and coworkers (2014) when evaluating the effect of surfactant concentration on NLC formulation properties.^29^ In which case the authors proposed that an excess of surfactant forms micelles that can agglomerate, leading to instabilities.^29,31^ In high lipid proportions, such as in zone 2, there is no excess surfactant. It is likely that a sufficient volume of lipid is necessary to form a surface area that accommodates all surfactants, with little or no remaining micelles, to achieve stability, as observed by Helgason et al.^30^

Good stability can also be observed when the TL:Sur ratio highly favors surfactants in zone 3. Smaller particles are achieved with higher concentrations of surfactants, in accordance with Witayaudom and Klinkesorn, who reported that increasing the concentration of surfactants resulted in a reduction of mean particle size in lipid nanoparticles.^11^ Pezeshki et al. also reported that when surfactant concentration is high enough, it reduces the tension between the lipid matrix and the aqueous phase to the point of reducing particle size.^29^ A particle size reduction results in a higher surface area to be covered by surfactant molecules,^11^ consuming what would otherwise be an excess of surfactants. According to the DoE model, intermediate values of the TL:Sur ratio would not present such stability, possibly for not presenting a surface area compatible with the amount of surfactant molecules.

Based on these findings, an optimized formulation was identified with a Liq:So ratio of 1:7 and a TL:Sur ratio of 1:3, with a predicted stability of over 28 days. This formulation demonstrated excellent particle size stability over 28 days. Three batches of the optimized formulation were prepared, and the particle size distribution was monitored for 28 days (Figure 3).

**Figure 3:**
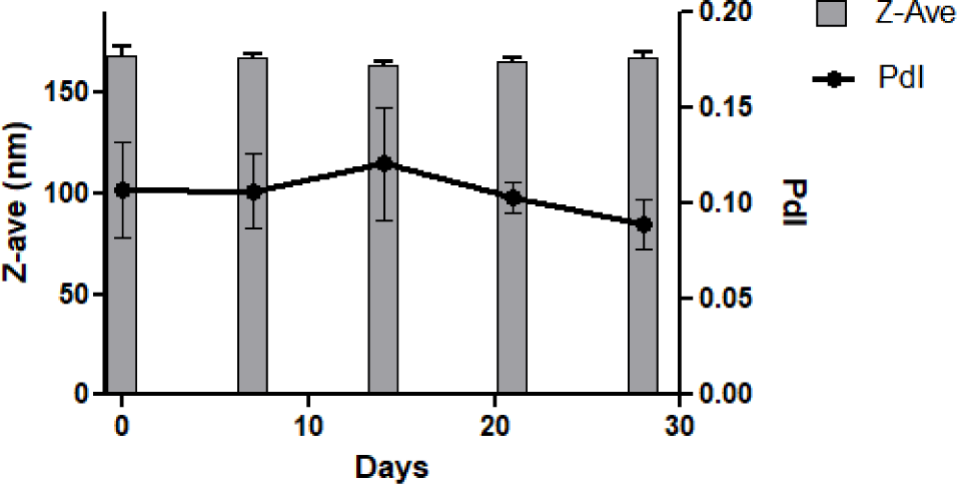
*Z*-average and polydispersity index of optimized formulation measured once a week during 28 days, represented by the mean of three separate batches and their standard deviation as the error bar.

One-way variance analysis (ANOVA) showed no statistical difference in the *Z*-average or PdI (*p* > 0.05) during the 28 days. In addition, all batches were stable for 28 days according to the parameters previously determined for Method 2: *Z*-average variation under 8%, PdI under 0.2, and the sample mean particle size did not deviate by more than 5.0% within 3 readings. This confirms that the role of the tested formulation parameters, liquid-to-solid lipid ratio and total lipids-to-surfactants ratio, on stability was significant and should be considered in the development of NLCs, and possibly other drug-delivery vehicles.

DoE is a valuable tool in the development of nanocarriers for drug delivery systems. Even though particle size stability is one of the most important qualities for nanoparticle development, it is mathematically challenging to establish this variable. This leads to a lack of control and understanding of the factors that really affect the stability of the developed nanoparticles. The obtained results indicate that the effect of independent variables on particle size stability can be predicted when using a time unit (days) and pre-established parameters to classify particles as stable or unstable at each time point. Therefore, this demonstrates the breaking of a paradigm regarding nanoparticle characterization and development. The use of particle size stability as a DoE response factor can accelerate the nanoparticle development. This approach is particularly valuable for future applications in drug delivery systems, where stability is crucial for pharmaceutical advancements.

## Supplementary Material



## Notes

Additional experimental details, materials, and methods (PDF)
