# Supplementary material for: Stability-Focused Nanoparticle Development: Applying Particle Size Stability as a Response Factor in the Design of Experiments
Source: ACS Omega. 2025 May 7;10(25):26546–50. doi: 10.1021/acsomega.5c00613 (PMC12223868; doi:10.1021/acsomega.5c00613)
Supplement: Supplementary file 1 [file ao5c00613_si_001.pdf]

SUPPORTING INFORMATION

Stability focused nanoparticle development: Applying particle size stability as a response factor in design of experiment

Vanessa V.B Muccelin[1], Otavio C. Vellozo[1], Thais L. Valente[1], Carolina G. Pupe[1], Edison L.S. Carvalho[1], Cassia B. Detoni[1]*

Formulation technology Research Group. Institute of Pharmaceutical Sciences – Federal University of Rio de Janeiro, Macaé-RJ, Brazil.

SI 1: Additional experimental details, materials, and methods

Materials

Tucumã butter (Amazon oil, Ananindeua – PA, Brazil); sorbitan monooleate (Span 80) (Lobachemie, Mumbai - India); polysorbate 80 (Tween 80) (Vetec, Duque de Caixias – RJ, Brazil); *Copaifera officinalis* resin oil (Amazon oil, Ananindeua – PA, Brazil); Acetone (Vetec, Duque de Caixias – RJ, Brazil); β-cariofileno (Sigma, Saint Louis-MO, USA).

Nanostructured lipid carrier preparation

In order to understand the influence of component proportion on nanostructured lipid carriers particle size stability a center composite, surface response experiment with two continuous predictors and center point sextuplicate was designed using Minitab Statistical Software. The predictores used were liquid to solid lipid ratio (Liq:So), at levels from 0.5:1 to 2:1, and proportion between total lipids and surfactants (TL:Sur) from 1:1 to 2.5:1. The solvent volume was kept the same, acetone 27 mL and water 53 mL, as was surfactant content, 0.04g of sorbitan monooleate and 0.04g of polysorbate 80, throughout all formulations. After solvent displacement, acetone was removed and volume was adjusted to 10 mL in a Rotavapor R-100 (Buchi). The program generated 14 experiments (Tabela S1), in which the reproducibility was tested through a center point sextuplicate.

The amount of copaiba oil and tucumã butter weighed in each run was calculated in grams per 10 mL (Table S1).

Table S1. Proportions and mass (g) of ingredients used to prepare 10 mL of each formulation.

Experiment n°	Liq:So	TL:Sur	TL* (g)	Tucumã Butter* (g)	Copaiba oil* (g)
1	2.000	1.000	0.080	0.0267	0.0533
2	0.189	1.750	0.140	0.1177	0.0223
3	1.250	2.810	0.225	0.0999	0.1249
4	0.500	1.000	0.080	0.0533	0.0267
5	1.250	1.750	0.140	0.0622	0.0778
6	1.250	1.750	0.140	0.0622	0.0778
7	1.250	1.750	0.140	0.0622	0.0778
8	1.250	1.750	0.140	0.0622	0.0778
9	1.250	0.690	0.055	0.0245	0.0306
10	0.500	2.500	0.200	0.1333	0.0667
11	1.250	1.750	0.140	0.0622	0.0778
12	1.250	1.750	0.140	0.0622	0.0778
13	2.000	2.500	0.200	0.0667	0.1333
14	2.311	1.750	0.140	0.0423	0.0977

* The mass demonstrated was used to prepare 10 mL of formulation.
** Every 10 mL of formulation had 0.04g of sorbitan monooleate and 0.04g of polysorbate 80.

Nanostructured lipid carrier Formulation analysis

Formulations were characterized according to z-average (z-ave), polydispersity index (PdI) and pH during 28 days (0,7,14,21 and 28) as seen in Table S3. Z-average and PdI was measured using dynamic light scattering (ZetaSizer NanoZS90 - Malvern). The samples were diluted in ultrapure water (MilliQ) (2:100) and analyzed immediately after dilution. Sample dilution was determined by evaluating tucumã butter NLC without copaiba oil (blanc NCL) diluted in ultrapure water at 1:100, 2:100, 3:100, 4:100 e 5:100, in triplicate. The dilution with lowest Cumulant Fit Error was chosen (Table S2).

Table S2. Dynamic light scattering analysis Cumulant Fit Error (mean and standard deviation of triplicate) of tucumã butter nanostructured lipid carrier at different dilution in water.

Cumulant Fit Error		
[C]	Mean	Standard deviation
1:100	5.35×10^{-4}	4.11×10^{-5}
2:100	5.16×10^{-4}	5.51×10^{-5}
3:100	5.96×10^{-4}	5.71×10^{-5}
4:100	5.48×10^{-4}	1.56×10^{-4}
5:100	7.71×10^{-4}	1.06×10^{-4}

PH was determined as an internal control to identify possible chemical alterations. Measurements were taken directly from the formulation (no dilution), descarting the first reading and considering the second (MS Tecnopon Equipamentos Especiais - LTDA). Formulations pH ranged from 5.8 to 4.5 during the entire experiment.

Table S3 - Z-average, polydispersion index (PdI) and pH of all 14 runs analyzed during 28 days.

Experiment n°		**Dia 0**	**Dia 7**	**Dia 14**	**Dia 21**	**Dia 28**
**1**	Z-average (nm)	153.1 ± 3.109	153 ± 1.473	144.2 ± 2.501	141.3 ± 2.023	140.5 ± 1.007
	PDI	0.126 ± 0.004	0.114 ± 0.014	0.112 ± 0.02	0.117 ± 0.021	0.107 ± 0.014
	pH	5.71	5.41	4.94	5.28	4.6
**2**	Z-average (nm)	176.7 ± 0.808	178.5 ± 2.403	172 ± 2.318	174.5 ± 0.6110	174.9 ± 4.782
	PDI	0.133 ± 0.008	0.144 ± 0.006	0.191 ± 0.011	0.140 ± 0.011	0.127 ± 0.018
	pH	5.26	5.41	5.43	5.29	4.79
**3**	Z-average (nm)	185.7 ± 2.146	183.3 ± 1.474	185.8 ± 1.601	182 ± 0.5132	2127 ± 813.2
	PDI	0.120 ± 0.014	0.129 ± 0.031	0.1 ± 0.015	0.137 ± 0.030	0.791 ± 0.232
	pH	5.72	5.74	5.4	5.23	5.58

**4**	Z-average (nm)	180.2 ± 2.318	182.5 ± 1.498	185.8 ± 5.408	179.8 ± 0.6245	177.6 ± 1.250
	PDI	0.108 ± 0.037	0.134 ± 0.008	0.128 ± 0.010	0.109 ± 0.039	0.121 ± 0.034
	pH	5.71	5.76	5.15	5.79	5.8
**5**	Z-average (nm)	122 ± 2.443	122.2 ± 0.8185	125 ± 4.536	123.9 ± 2.458	107.9 ± 1.650
	PDI	0.119 ± 0.013	0.135 ± 0.009	0.125 ± 0.027	0.142 ± 0.007	0.133 ± 0.009
	pH	5.72	5.8	4.81	4.53	4.52
**6**	Z-average (nm)	157.2 ± 2.307	157 ± 2.946	154.3 ± 1.873	154.8 ± 1.380	159.8 ± 4.122
	PDI	0.145 ± 0.002	0.143 ± 0.023	0.157 ± 0.022	0.139 ± 0.006	0.133 ± 0.008
	pH	5.65	5.5	5.13	4.62	5.15
**7**	Z-average (nm)	177.6 ± 1.102	185 ± 4.007	181.6 ± 1.353	285.5 ± 1.589	133.4 ± 1.868
	PDI	0.133 ± 0.012	0.134 ± 0.009	0.121 ± 0.031	0.926 ± 0.063	0.121 ± 0.020
	pH	5.73	5.89	5.33	5.72	5.68
**8**	Z-average (nm)	214.7 ± 0.8505	213.3 ± 2.255	220.5 ± 8.238	251.4 ± 1.277	193.8 ± 2.290
	PDI	0.115 ± 0.013	0.114 ± 0.031	0.082 ± 0.032	0.121 ± 0.016	0.109 ± 0.021
	pH	5.55	5.59	5.35	5.43	5.15
**9**	Z-average (nm)	184.3 ± 2.203	181.9 ± 2.684	186.1 ± 2.597	180.1 ± 2.434	130.7 ± 1.305
	PDI	0.113 ± 0.014	0.101 ± 0.021	0.124 ± 0.019	0.117 ± 0.031	0.142 ± 0.027
	pH	5.65	5.58	5.66	5.43	5.2
**10**	Z-average (nm)	234.2 ± 4.689	235.4 ± 4.729	231.2 ± 2.219	234.9 ± 3.889	235.2 ± 1.664
	PDI	0.120 ± 0.002	0.090 ± 0.028	0.131 ± 0.019	0.118 ± 0.020	0.332 ± 0.023
	pH	5.31	5.41	4.92	4.77	4.61

**11**	Z-average (nm)	203.8 ± 2.344	207.6 ± 4.951	204.4 ± 2.470	120.3 ± 0.6	117.7 ± 10.91
	PDI	0.137 ± 0.037	0.152 ± 0.021	0.122 ± 0.017	0.381 ± 0.032	0.180 ± 0.568
	pH	5.26	5.72	5.54	5.26	5.49
**12**	Z-average (nm)	186.3 ± 1.637	189.5 ± 3.808	185.9 ± 2.517	133.3 ± 6.504	134.2 ± 3.053
	PDI	0.120 ± 0.018	0.090 ± 0.028	0.141 ± 0.018	0.587 ± 0.094	0.113 ± 0.063
	pH	5.63	5.63	4.96	4.98	5.15
**13**	Z-average (nm)	223.5 ± 3.650	223.1 ± 3.318	221.2 ± 1.890	221.8 ± 1.803	217.5 ± 5.260
	PDI	0.152 ± 0.025	0.134 ± 0.023	0.162 ± 0.017	0.140 ± 0.023	0.135 ± 0.021
	pH	5.2	5.28	4.75	4.88	5.31
**14**	Z-average (nm)	187.5 ± 0.8327	189.3 ± 1.332	186.6 ± 1. 097	183.8 ± 3.288	129 ± 1.601
	PDI	0.118 ± 0.012	0.133 ± 0.032	0.132 ± 0.006	0.125 ± 0.011	0.073 ± 0.057
	pH	5.65	5.54	5.19	4.74	4.71

Experimental design methodology

In this research, two possible stability response factors were used to determine particle size stability for the obtained NLC. In Method 1 stability of NLC was measured in a size unit, nanometers, considering particle size variation after 28 days (ΔZ-average). In Method 2 stability of NLC was measured in a time unit such as days. To define if a formulation was stable at a certain time, 3 parameters were considered Z-average variation under 10%, PdI under 0.2, and the sample mean particle size did not deviate over 5.0% within 3 readings. Minitab® software performed surface response analysis and graphical representation (Figure S1 and Figure S2). Method 2 showed a better fit to the model (Table S5) than method 1 (Table S4) and the Regression Equation is shown below (Eq. S1).

$$Stability = 54{,}2 - 22{,}56\ (Liq:So) - 35{,}8\ (TL:Sur) + 11{,}67\ (Liq:So)^2 + 11{,}67\ (TL:Sur)^2 - 3{,}11\ (Liq:So) \times (TL:Sur)$$

(Eq. S1)

Where, TL:Sur is the proportion between total lipids and surfactants; So is tucumã butter (solid lipid); Liq is copaiba oil (liquid lipid) and Liq:So is the proportion between liquid and solid lipids.

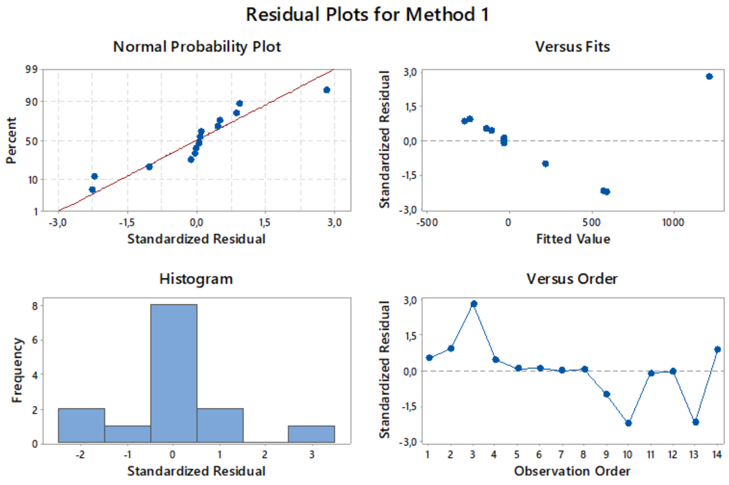

Figure S1. Residual plots for Method 1 in which stability of NLC was measured in a size unit, nanometers, considering particle size variation after 28 days (ΔZ-average).

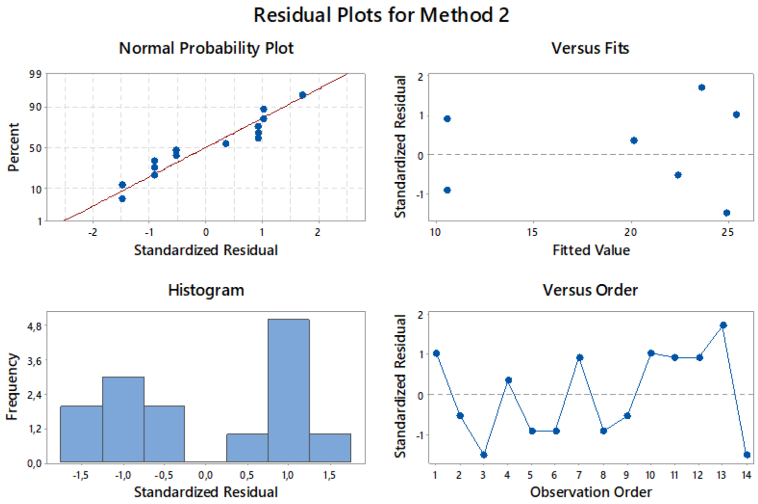

Figure S2. Residual plots for Method 2 in which stability of NLC was measured in days.

Table S4. Analysis of Variance for Response Surface Regression: Z-average variation versus Liq:So; TL:Sur (Method 1).

Response Surface Regression: Z-ave variation versus Liq:So; TL:Sur

Analysis of Variance

Source	DF	Adj SS	Adj MS	F-Value	P-Value
Model	5	2185981	437196	2,46	0,124
Linear	2	999747	499873	2,81	0,119
Liq:So	1	1310	1310	0,01	0,934
TL:Sur	1	998436	998436	5,62	0,045
Square	2	1186218	593109	3,34	0,088
Liq:So*Liq:So	1	92454	92454	0,52	0,491
TL:Sur*TL:Sur	1	1039062	1039062	5,85	0,042
2-Way Interaction	1	17	17	0,00	0,992
Liq:So*TL:Sur	1	17	17	0,00	0,992
Error	8	1420765	177596		
Lack-of-Fit	3	1416108	472036	506,81	0,000
Pure Error	5	4657	931		
Total	13	3606746			

Table S5. Analysis of Variance for Response Surface Regression: Stability (days) versus Liq:So; TL:Sur (Method 2).

Analysis of Variance

Source	DF	Adj SS	Adj MS	F-Value	P-Value
Model	5	615,125	123,025	6,99	0,009
Linear	2	12,250	6,125	0,35	0,716
Liq:So	1	6,125	6,125	0,35	0,572
TL:Sur	1	6,125	6,125	0,35	0,572
Square	2	590,625	295,313	16,77	0,001
Liq:So*Liq:So	1	318,029	318,029	18,06	0,003
TL:Sur*TL:Sur	1	318,029	318,029	18,06	0,003
2-Way Interaction	1	12,250	12,250	0,70	0,428
Liq:So*TL:Sur	1	12,250	12,250	0,70	0,428
Error	8	140,875	17,609		
Lack-of-Fit	3	67,375	22,458	1,53	0,316
Pure Error	5	73,500	14,700		
Total	13	756,000			

Furthermore, the software, using given factors and limits, determined an optimized formulation with maximized stability (Figure S3).

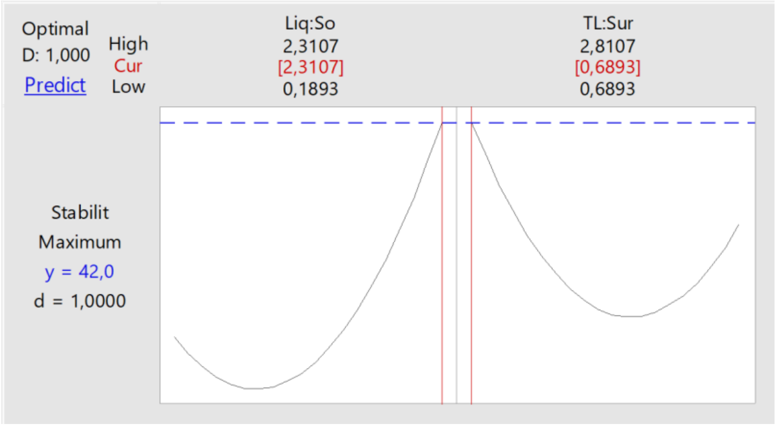

Figure S3. Optimization plot for Method 2 in which stability of NLC was measured in days.
